# Serum HBV RNA quantification: useful for monitoring natural history of chronic hepatitis B infection

**DOI:** 10.1186/s12876-019-0966-4

**Published:** 2019-04-16

**Authors:** Yayun Liu, Meng Jiang, Jianya Xue, Hongli Yan, Xuesong Liang

**Affiliations:** 1 Department of Infectious Diseases, Changhai Hospital, Second Military Medical University, 168 Changhai Road, Shanghai, 200433 China; 2 Department of Reproductive Medicine Center, Changhai Hospital, Second Military Medical University, 168 Changhai Road, Shanghai, 200433 China

**Keywords:** Hepatitis B virus (HBV), Pregenomic RNA (pgRNA), Natural history, Hepatitis B e antigen (HBeAg), Hepatitis B surface antigen (HBsAg)

## Abstract

**Background:**

As an alternative biomarker of intrahepatic covalently closed circular DNA (cccDNA) transcriptional activity, hepatitis B virus (HBV) RNA may evolve during long-lasting virus-host interactions during chronic hepatitis B viral infection. The distribution pattern of serum HBV RNA levels in the natural course of chronic HBV infection remains unclear. The aim of this study was to evaluate the levels of HBV RNA during the natural course of CHB and the role in distinguishing the natural history of HBV infection.

**Methods:**

A total of 291 treatment-naïve chronic HBV carriers were enrolled. Based on the clinical, biochemical, serological, and histological data as well as HBV DNA levels, patients were classified into the following four categories: the immune-tolerant phase (IT,*n* = 35), HBeAg-positive immune-active phase (EPIA,*n* = 121), inactive chronic hepatitis B(ICH,*n* = 58) and HBeAg-negative immune reactive hepatitis (ENH,*n* = 77). The parameters and distribution patterns of serum HBV RNA were evaluated in relation to viral replication status, immune phase, disease category and Child-Pugh class. The relationships between serum HBV RNA and other serum hepatitis B viral markers were also analyzed.

**Results:**

Serum HBV RNA levels were significantly lower in the HBeAg-negative patients compared to those in the HBeAg-positive patients, with the lowest levels seen in inactive carriers. In HBeAg-negative patients, serum HBV RNA levels increased if there is reactivation to active hepatitis and showed obvious superiority for the combination of serum HBV DNA (cutoff>3.39 Log copies/mL) and HBsAg (cutoff>2.74 Log IU/mL) in discriminating between ‘HBeAg-negative immune reactive’ phase and inactive chronic hepatitis B phases of HBeAg-negative chronic HBV infection. Serum HBV RNA levels were positively correlated with serum HBV DNA and HBsAg levels in all chronic HBV-infected patients. A stratified analysis revealed that a correlation between serum HBV RNA and HBV DNA or HBsAg was present in HBeAg-positive patients; however, in HBeAg-negative patients, serum HBV RNA was positively correlated with HBV DNA only.

**Conclusion:**

During the natural course of chronic HBV infection, serum HBV RNA levels vary. Serum HBV RNA can act as a biomarker to predict the natural history of disease in chronic hepatitis B patients. In treatment-naïve HBeAg-negative chronic HBV-infected individuals, serum HBV RNA shows superiority in differentiating the ‘HBeAg-negative reactive’ phase.

## Background

Chronic hepatitis B viral infection is a global health problem, especially in the Asian-Pacific region. Approximately 2 billion people are chronically infected with hepatitis B virus (HBV) worldwide and about 650,000 people die annually from HBV infection related liver failure, liver cirrhosis and liver cancer [1, 2]. Although the first line antiviral agents such as nucleos(t) ide analogues (NAs) can efficiently inhibit HBV replication and control disease progression in almost all patients, these drugs rarely lead to elimination of chronic HBV infection completely due to their little effect on covalently closed circular DNA(cccDNA),which is believed to be the main cause of viral persistence [3–6]. Accordingly, rebounding of the virus after drug withdrawal is common [7]. As the main cause of HBV persistence and drug resistance, monitoring of intrahepatic cccDNA level is of great significance for evaluating antiviral therapy efficacy and estimating treatment endpoint [8]. However, in actual clinical practice, intrahepatic cccDNA monitoring is not easy to be widespread due to need liver biopsy, inter-observer variability and potential complications [9, 10]. Therefore, surrogate markers for intrahepatic cccDNA transcriptional activity are required.

HBV DNA, RNA, and hepatitis B surface antigen (HBsAg) levels have been confirmed to be positively correlated with intrahepatic cccDNA transcription activity in chronic HBV-infected individuals, especially those with hepatitis B e antigen (HBeAg)-positive; therefore, these markers are important surrogates for indicating intrahepatic cccDNA transcriptional activity [11–18]. In recent years HBsAg quantitative detection methods have been commercialized and have been widely used in antiviral efficacy prediction [19–26] and the management of treatment-naïve chronic HBV-infected individuals [27–29]. However, many factors may be obstacles for the use of serum HBsAg as a surrogate for intrahepatic cccDNA transcription activity with long-term NAs treatment, such as HBV DNA integration [30], long-term NAs treatment related HBsAg retention [31].Unlike HBsAg, HBV RNA is derived only from intrahepatic cccDNA, and its quantification is not affected by viral antigens or antibody complexes. Therefore, the serum HBV RNA level can more accurately reflect intrahepatic cccDNA transcriptional activity. Despite the usefulness of serum HBV RNA quantification in the management of chronic HBV infection, especially in predicting antiviral efficacy [15, 16] and viral rebounding after drug withdrawal as well as predicting viral resistance [17, 32], there is currently a lack of corresponding data on the baseline level of serum HBV RNA during the natural course of CHB. Hence, the aim of this cross-sectional study was to evaluate the levels of HBV RNA in the consecutive phases of HBV infection in patients not receiving antiviral therapy and to evaluate the significance of HBV RNA quantification in monitoring the natural history of chronic HBV infection.

## Methods

### Patients

A cross-sectional study was performed in 291 treatment-naïve patients with chronic HBV infection from Shanghai Changhai hospitals.

All patients tested positive for HBsAg more than 6 months, negative for markers of the hepatitis C virus (HCV), hepatitis D virus (HDV), and human immunodeficiency virus (HIV). Markers such as ceruloplasmin, anti-nuclear antibodies and anti-mitochondrial antibodies for co-existent autoimmune and metabolic liver diseases were negative. Patients with hepatocellular carcinoma (HCC) and acute or acute-on-chronic liver failure were excluded. Patient demographics, medical history, liver biochemistry, quantitative HBsAg (qHBsAg) and HBeAg status were determined. HBV DNA levels in these patients were obtained when the serum samples were collected.

The patients were classified into a phase of CHB after a follow-up period of 3–6 months. The phase of CHB in each patient was determined by HBeAg/anti-HBe sero-status as well as measurements of HBV DNA and serum alanineamino transferase (ALT) levels and liver imaging findings and medical history data according to the recently published clinical practice guidelines of the American Association for the Study of Liver Diseases(AASLD) [33].

The study was conducted according to the guidelines of the Declaration of Helsinkiand was approved by the Shanghai Changhai hospital ethics research committee (CHEC2017–118). Written informed consent was obtained from each patient.

### HBV RNA quantification

The HBVRNA was detected by the RNA simultaneous amplification testing method(HBV-SAT) based on real-time fluorescence detection of RNA transcription-mediated nucleic acid amplification using the HBV-SAT kit (Rendu biotechnology, Shanghai, China). Primers and probes were designed to amplify HBV pre-genome RNA (pgRNA) conserved region [34]]. One primer complementary to the target RNA template included a T7 promoter sequence on the 5′ end. The RNA beacon probe was labeled with FAM at the 5′ end and DABCYL at the 3’end. RNA from 250 μL serum was extracted using magnetic micro-particles with HBV pgRNA-specific oligonucleotides. The magnetic micro-particles with the extracted target RNA were added to the reaction system with Moloney murine leukemia virus (MMLV) reverse transcriptase as well as T7 RNA polymerase primers and probes in a proprietary reaction mixture. RNA extraction, amplification and detection were processed on an automated Auto SAT system (Rendu biotechnology, Shanghai, China). When the probe binds to the amplicon, the probe emits a fluorescent signal at a specific wave length when excited by a light source. As more probes hybridize to the amplicon, a higher fluorescent signal is generated. The time taken for the fluorescent signal to reach a specified threshold is proportional to the starting HBV RNA concentration. The efficiency of RNA extraction and amplification for each sample was validated by the detection of an armored RNA internal control added during sample lysis. Calibration of the HBV RNA assay was performed using an armored HBV RNA standard traceable to HBV RNA standard in vitro transcripts.

### Evaluation of HBV-SAT performance

According to the HBV-SAT kit instructions, the limit of HBV pgRNA detection (LOD) is 100copies/mL. The assay sensitivity and linearity were evaluated on a clinical serum specimen with a high concentration of pgRNA (8.04 ± 0.12 log copies/mL) detected with a previously described method [17]. The assay sensitivity was determined by diluting the clinical serum specimen to 50copies/mL, and then a10-folddilutionseriesoftheclinicalserumspecimenfrom2.0 log copies/mL to 8.0 log copies/ml was quantitated to determine linearity. Each dilution was tested in triplicate. The assay specificity was evaluated using the serum from 30 HBV-negative blood donors.

### Serum HBsAg quantification

Serum HBsAg levels were measured by using commercially available kits (Abbot Laboratories, North Chicago, IL) in our clinical lab. The dynamic range was 0.05–250 IU/mL. The samples were diluted 1:500or 1:1000 using the ARCHITECT HBsAg Manual Diluent (Abbott Diagnostics) if> 250 IU/mL.

### HBV DNA measurement

Serum HBV DNA was quantified by using the Determination Kit for hepatitis B viral DNA (Sansure Biotech, China), with a detection range of 5 × 10^2^copies/mL-5 × 10^8^copies/mL.

### Statistical analysis

All non-normal distribution data were presented as the median and interquantile range (IQR) and normal distribution data were presented as mean ± SD. The variables were compared between groups using the Mann-Whitney U and Fisher’s exact tests for univariate comparisons as well as the Kruskall-Wallis test and ANOVA for multivariate comparisons. The regression and Spearman correlation coefficients(r) were used to compare the correlation between two variables. Receiver operating characteristic (ROC) curves and area under the ROC curves (AUROC) were used to estimate diagnostic performance. The best cutoff for maximal diagnostic accuracy was selected based upon the highest sum of sensitivity and specificity. A *P*-value (2-tailed) of 0.05 was considered statistically significant. All statistical analyses were performed using SPSS software (ver.21.0.0; Chicago, IL,USA).

## Results

### Baseline characteristics

A total of 378 treatment-naïve chronic HBV-infected individuals were recruited for the study from March 2017 to Nov 2017. In the end, 291 patients were enrolled, and 87 patients were excluded because of co-infection with HCV (*n* = 5), EB (*n* = 2), or CMV (*n* = 3) or because of incomplete data (*n* = 77). The median (interquartile range, IOR) age was 37(30, 46) years. There were 135HBeAg-negative CHB patients and 156 HBeAg-positive CHB patients. The HBeAg-positive CHB patients were significantly younger, with a median (IQR) age of 33(28,41) years compared with 41(33,52) for the HBeAg-negative CHB patients (t = 5.924,*P* = 0.000). The HBeAg-positive CHB group had a higher median(IQR) ALT of 88.5(42.5261.0)IU/L,AST of 58.5(31,116)IU/L, HBV DNA level of 7.65(5.87,8.43) log copies/mL and qHBsAg level of 3.92 (3.27,4.56) log IU/mL compared with the HBeAg-negative patients with ALT [36(20.5100.5) IU/L], AST[27 (21,56) IU/L], HBV DNA[3.16 (2.74,5.20)log copies/mL] and qHBsAg [2.98(2.38,3.58) log IU/mL]. Of the HBeAg-positive CHB patients, 35(22.4%) were in the IT phase, and the remaining 121(77.6%) were in the EPIA phase. Of the HBeAg-negative CHB patients, 77(57.0%) and 58(43.0%) were in the ICH and ENH phases, respectively. The baseline characteristics of the 291 patients are summarized in Table 1.

**Table 1:** Baseline population characteristics

Parameters	Chronic HBV infection							
	Total (*n* = 291)	HBeAg positive			HBeAg negative			*P*^#^ value
		Total (*n* = 156)	Immune tolerant, IT (*n* = 35)	HBeAg positive immune active hepatitis, EPIA (*n* = 121)	Total (*n* = 135)	Inactive chronic hepatitis B, ICH (*n* = 77)	HBeAg negative reactive hepatitis, ENH (*n* = 58)	
Age, years median(IQR)	37(30,46)	33(28,41)	30(27.5,34.0)	34.0 (28.0,42.0)	41(33,52)	40.0 (31.0,50.0)	42.0(35.0,53.0)	0.000
Gender, M/F	206/85	114/42	25/10	89/32	92/43	45/32	47/11	0.028
Disease category(CH/LC/HCC)	240/51/0	125/31/0	30/0/0	90/31/0	115/20/0	74/3/0	41/17/0	/
Child-Pugh class(A/B/C)	31/11/9	23/4/4	na	23/4/4	8/7/5	1/1/1	7/6/4	/
HBsAg, log IU/mL, median(IQR)	3.49(2.91,4.12)	3.92(3.27,4.56)	4.56(4.13,4.71)	3.71(3.22,4.31)	2.98(2.38,3.58)	2.94(2.32,3.54)	3.00(2.48,3.66)	0.000
HBeAg, log S/CO median(IQR)	na	2.81(1.67,3.11)	3.01(3.07,3.17)	2.58(1.46,3.03)	na	na	na	0.005
HBV DNA,log copies/mL, median(IQR)	5.94(3.63,8.14)	7.65(5.87,8.43)	8.44(8.15,8.43)	7.11(5.39,8.44)	3.61(2.74,5.20)	2.74(2.74,3.82)	5.48(4.09,6.65)	0.000
HBV RNA,log copies/mL median(IQR)	5.50(2.57,7.16)	6.82(5.57,7.98)	7.87(6.08,8.30)	6.67(5.433,7.80)	2.40(2.00,5.23)	2.00(2.00,3.04)	5.31(3.57,6.37)	0.000
ALT,IU/L median(IQR)	59(30,191)	88.5(42.5261.0)	34(28.5,43.5)	153(66.50,348.50)	36(20,100.50)	24(18,34)	131(67,309)	0.000
AST,IU/L median(IQR)	41(23,102)	58.5(31,116)	27(23,31.5)	76.5(44,157)	27(21,56)	21(19,26)	89.5(39,188)	0.000

*IQR* interquantile range, *HBeAg* Hepatitis B e antigen, *CH* chronic hepatitis, *LC* liver cirrhosis, *na* not applicable, *HCC* hepatocellular carcinoma, *HBsAg* Hepatitis B surface antigen, *HBV* hepatitis B virus, *AST* aspartate aminotransferase, *ALT* alanine aminotransferase. *P#* value: compared between patients with HBeAg-positive and patients with HBeAg-negative

### Distribution of serum HBV RNA levels in various phases of chronic HBV infection

**Fig. 1:**
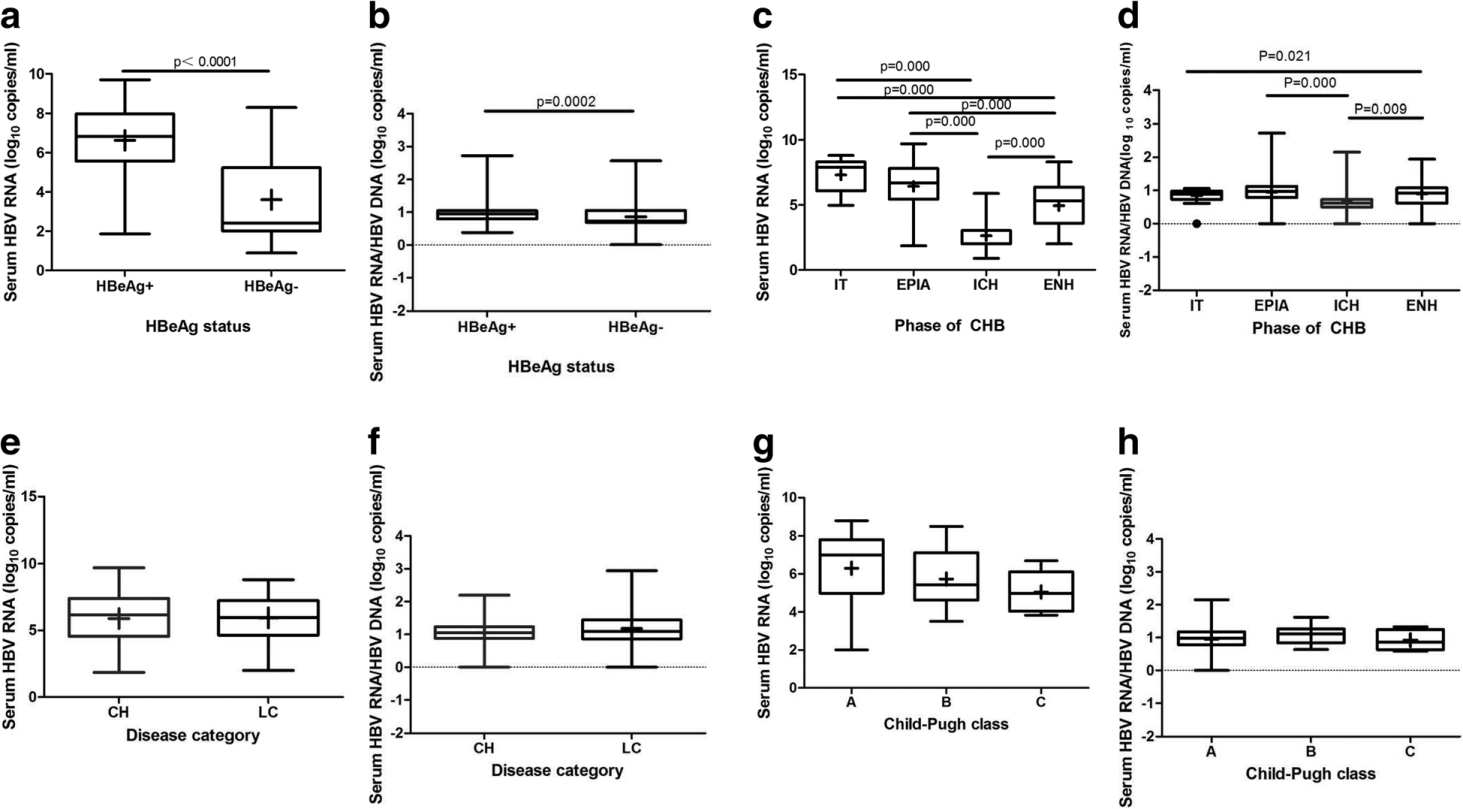
Distribution of serum HBV RNA titers throughout the natural course of CHB. Median values with 95% confidence interval (of median) represented. IT, immune tolerant;EPIA, HBeAg-positive immune-active phase; ICH, Inactive chronic hepatitis B phase; ENH, HBeAg-negative chronic hepatitis B phase;CH,chronic hepatitis;LC, liver cirrhosis

All ten repeated LOD testing results were positive for the pgRNA assay by HBV-SAT. The R^2^ value of the linear equation of the HBV-SAT results and theoretical concentration value was 0.9785. The assay specificity was confirmed by the negative results of serum from 30 HBV-negative blood donors. Serum HBV RNA levels were compared between subgroups stratified by viral replication status, natural phases, disease category and Child-Pugh class, as shown in Fig. 1. The serum HBV RNA values in the HBeAg-positive group were significantly higher than those in the HBeAg-negative group (median 6.82 Vs 2.40, log copies/mL, *P* < 0.0001; Fig. 1a).

With respect to the natural phases of chronic HBV infection, there were significant differences in the serum HBV RNA values among patients in the different phases (IT 7.87, EPIA 6.67, ICH 2.00 and ENH 5.31 log copies/mL, Fig. 1c). The ICH group had the lowest serum HBV RNA values compared to those of the other groups, and these values were significantly lower than those of another HBeAg-negative phase, ENH. In the assessment of patients in the EPIA and ENH phases, serum quantitative HBV RNA levels in patients with CHB and cirrhosis were 6.17(4.55,7.40) and 5.96(4.65,7.23) log copies/mL, respectively, showing a slight downward trend but with no significance (*P* = 0.871). The levels of HBV RNA in cirrhosis patients with Child-Pugh classes A, B and C were 7.00 (4.98, 7.80), 5.43 (4.62, 7.12) and 4.98 (4.03, 6.11) log copies/mL, respectively. The HBV RNA levels were not significantly different between the Child-Pugh classes (*P* = 0.08) (Fig. 1 e and g).

To assess the reverse transcriptional efficiency of pgRNA in the various chronic HBV infection phases, the ratio of serum HBV RNA to HBV DNA was calculated. As shown in Fig. 1b and d, the ratio of serum HBV RNA to HBV DNA in the HBeAg-positive group was remarkably higher than that of the HBeAg-negative groups, and moreover, this ratio in the ICH phase was lower than that in the other three phases (IT 0.99, EPIA 1.07, ICH 0.85 and ENH 1.04) but only significantly lower than that in the EPIA and ENH phases.

### Serum HBV RNA level was positively correlated with serum HBV DNA and HBsAg level in chronic HBV-infected individuals

Overall, the level of serum HBV RNA was positively correlated with serum HBV DNA (Spearman *r* = 0.793,*P* = 0.000) and HBsAg (Spearman *r* = 0.552,*P* = 0.000) in 291 1treatment-naïve chronic HBV-infected individuals. Stratified analysis revealed that the correlation between serum HBV RNA and DNA or HBsAg was presentin HBeAg-positive patients (RNA to DNA, *r* = 0.560, *P* = 0.000; RNA to HBsAg, *r* = 0.455,*P* = 0.000). However, in HBeAg-negative patients, serum HBV RNA was only positively correlated with HBV DNA (*r* = 0.639,*P* = 0.000).

**Fig. 2:**
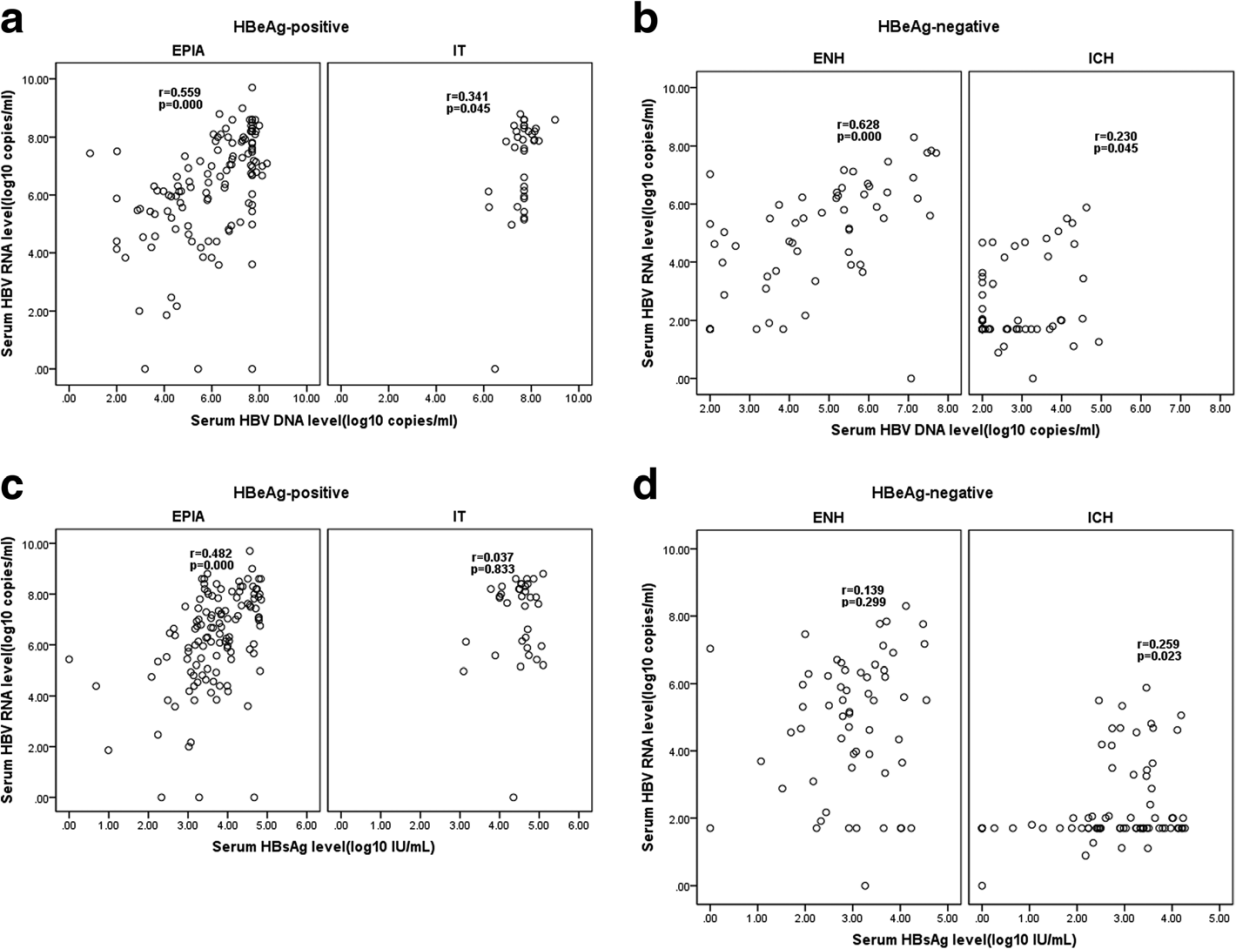
Correlation between serum HBV RNA titers and HBV DNA in various phases of CHB. IT, immune tolerant;EPIA, HBeAg-positive immune-active phase; ICH, Inactive chronic hepatitis B phase; ENH, HBeAg-negative chronic hepatitis B phase

A further stratified analysis of HBeAg-positive and-negative patients revealed that, in the EPIA phase, serum HBV RNA levels were moderately correlated with both serum HBV DNA and HBsAg levels (*r* = 0.559 versus 0.482,*P* = 0.000); however, in the IT phase, there was only a weak correlation between serum HBV RNA and HBV DNA (*r* = 0.341,*P* = 0.045) (Fig. 2a and c). In HBeAg-negative individuals, serum HBV RNA was found to be strongly correlated with HBV DNA level only in the ENH phase (*r* = 0.629,*P* = 0.000);however, in the ICH phase, there was only a weak correlation between both HBV DNA (*r* = 0.230,*P* = 0.045) and HBsAg (*r* = 0.250,*P* = 0.023) with HBV DNA (Fig. 2 b and d).

### Value of serum HBV RNA in monitoring chronic HBV infection

**Fig. 3:**
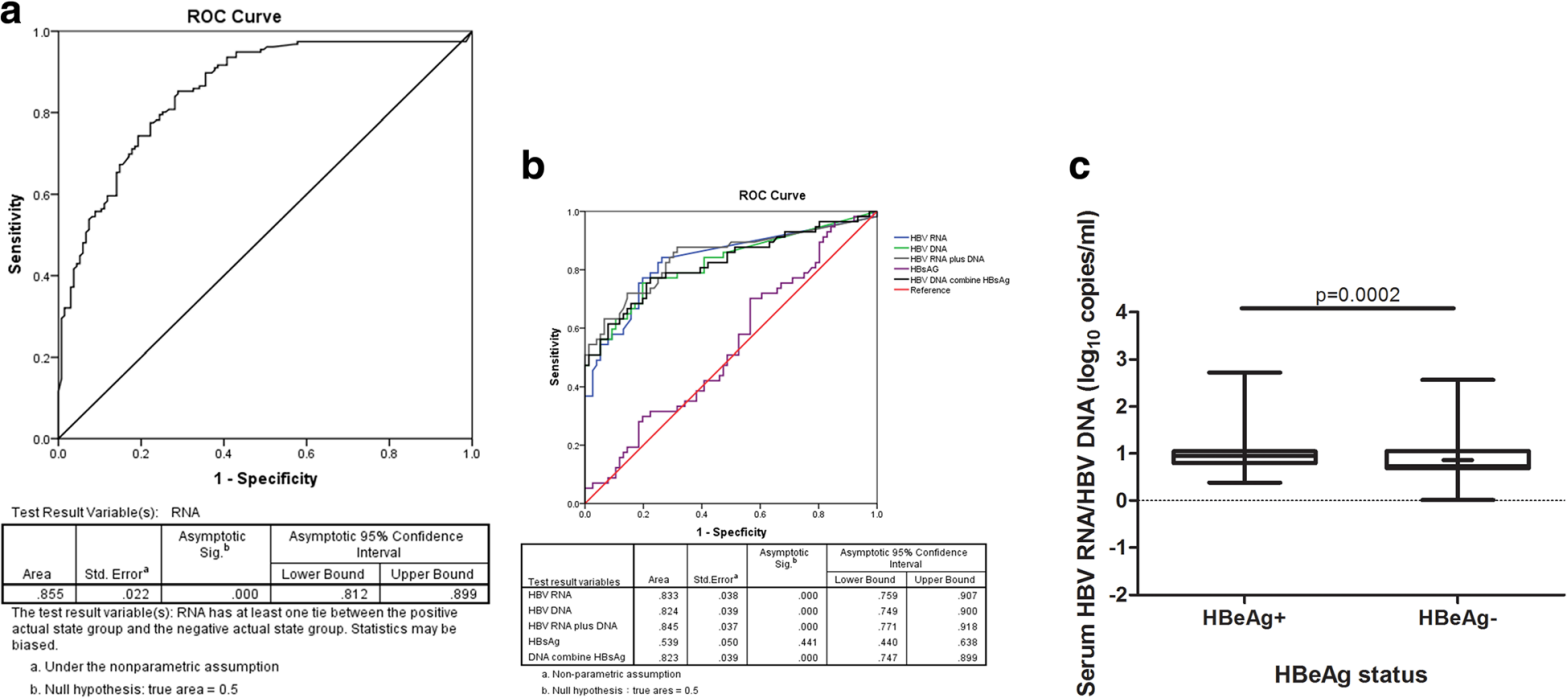
Receiver operating characteristic curve showing the diagnostic value of serum HBV RNA for HBeAg-positive status in chronic HBV infection and ‘aggressive HBeAg-negative CHB’ in HBeAg-negative chronic hepatitis B patients. A:serum HBV RNA level predicts HBeAg-positive CHB in chronic HBV-infected individuals; B:viral markers differentiate ‘aggressive HBeAg-negative CHB’ in HBeAg-negative chronic hepatitis B patients

Serum HBV RNA levels are obviously related to HBeAg status during the natural course of chronic HBV infection. HBV RNA levels tend to decrease when progresses to HBeAg-negativity. When an ROC curve was drawn to predict HBeAg-positive status based on the serum HBV RNA value, the AUROC was 0.855, *P* = 0.000, 95% confidence interval (CI) (0.812,0.899). (Fig. 3a).

Although HBV RNA levels tend to decrease when progresses to HBeAg-negativity and inactivity, these levels increase if there is reactivation to active hepatitis, as shown in Fig. 1c.Considering the significant difference in serum HBV RNA levels in HBeAg-negative patients in various phases, we evaluated the potential utility of serum HBV RNA in discriminating between the ‘inactive carrier’ and ‘HBeAg-negative CHB’. The ROC curve was drawn, and the AUROC was 0.833, 95% CI 0.759 to 0.907, *P* = 0.000 (Fig. 3b). At a cutoff of 2.11 log copies/mL, the serum HBV RNA level predicted HBeAg-negative CHB phase with 78.19% accuracy, 84.21% sensitivity, 73.68% specificity,70.59% positive predictive value (PPV) and 86.15% negative predictive value (NPV) (Table 2). In addition, serum HBV RNA levels showed obvious superiority for the combination of serum HBV DNA (cutoff>3.39 log copies/mL) and HBsAg (cutoff>2.74log IU/mL) in discriminating between these two phases of HBeAg-negative chronic HBV infection (Table 2).

**Table 2:** Prediction of hepatitis B virus reactivation in HBeAg-negative chronic HBV infection by serum HBV RNA levels

Variables	HBV RNA >2.11(log *copies/mL*)	HBV DNA >3.39(log *copies/mL*)	HBsAg>2.74(log *IU/mL*)	HBV RNA >2.11(log copies/mL) & HBsAg>2.74(log *IU/mL*)	HBV DNA >3.39(log copies/mL) & HBsAg>2.74(log *IU/mL*)
Sensitivity(%)	84.21	75.4	70.17	59.64	56.14
Specificity(%)	73.68	80.26	42.11	81.58	86.84
PPV(%)	70.59	74.13	47.62	70.83	76.19
NPV(%)	86.15	81.33	65.31	72.94	72.52
Diagnostic accuracy(%)	78.19	78.19	54.13	72.18	73.68
*P* value*		*χ*^*2*^ = 1.362 *P* = 0.243	*χ*^*2*^ = 3.189 *P* = 0.074	*χ*^*2*^ = 8.515 *P* = 0.004	*χ*^*2*^ = 10.729 *P* = 0.001
*P* value^#^		*χ*^*2*^ = 0.928 *P* = 0.335	*χ*^*2*^ = 15.545 *P* = 0.000	*χ*^*2*^ = 1.364 *P* = 0.243	*χ*^*2*^ = 4.153 *P* = 0.042

*HBV* hepatitis B virus, *HBsAg* Hepatitis B surface antigen, *PPV* positive predictive value, *NPV* negative predictive value;*P value of the sensitivity comparison of RNA and other viral markers; ^#^*P* value of the specificity comparison of RNA and other markers; HBV RNA, HBV DNA and HBsAg cutoff value were obtained from ROC curve analysis

### Correlation between serum HBV RNA and clinical parameters

Serum HBV RNA was correlated with ALT and AST levels in both host immune active phases, EPIA and ENH. Moreover, in the EPIA phase, serum HBV RNA was negatively correlated with age (*r* = − 0.205, *P* = 0.024) and positively correlated with HBeAg level (*r* = 0.291,*P* = 0.001) (Table 3).

**Table 3:** Correlation between serum HBV RNA and laboratory parameters in different phases of chronic HBV infection

	IT (*n* = 35)		EPIA (*n* = 121)		ICH (*n* = 77)		ENH (*n* = 58)	
	r	*P* value	r	*P* value	r	*P* value	r	*P* value
Age(year)	−0.019	0.916	−0.205	0.024	−0.136	0.240	−0.054	0.689
ALT(*U/L*)	0.051	0.773	0.220	0.015	0.123	0.286	0.489	0.000
AST(*U/L*)	0.286	0.096	0.231	0.011	0.336	0.003	0.546	0.000
HBeAg(*S/CO*)	−0.215	0.214	0.291	0.001	/	/	/	/

*IT* immune tolerant, *EPIA* HBeAg-positive immune-active phase, *ICH* Inactive chronic hepatitis B phase, *ENH* HBeAg-negative chronic hepatitis B phase, *ALT* alanine aminotransferase, *AST* aspartate aminotransferase, *HBeAg* Hepatitis B e antigen

## Discussion

As a clinical surrogate marker for the transcriptional activity of cccDNA in the liver, HBV RNA has received increasing attention in recent years [15, 16, 35, 36]. Specifically, since Professor Lu Fengmin’s team determined that peripheral blood HBV RNA is pgRNA in 2016 [17], there have been increasing numbers of studies on peripheral blood HBV RNA levels in the management of chronic HBV infection [36–38]. In this study, we quantitated HBV RNA in 291 treatment-naïve chronic HBV-infected individuals by using the HBV-SAT assay. The RNA-SAT method combined with an automated Auto SAT system was used to complete a series of automated operations, including RNA extraction, amplification and quantitation, without the need to open the reaction tube lid, which minimized the risk of contamination and was highly specific and sensitive. SAT has been widely applied in clinical tests, such as those for *Mycobacterium tuberculosis* [39], entero virus [40] and sexually transmitted diseases.

In this study we revealed the changing patterns of serum HBV RNA levels over the natural history of HBV infection through investigating the serum level of HBV RNA in a large treatment naïve HBV infected individuals. During the natural history of chronic HBV infection, serum HBV RNA levels and HBV RNA to DNA ratios change significantly. The serum HBV RNA level and ratio of RNA to DNA were obviously higher in HBeAg-positive patients compared with those in HBeAg-negative patients. Stratified analysis revealed that serum HBV RNA level and the ratio of RNA to DNA in inactive carriers were the lowest compared to those in the other three phases. These results demonstrate that the virus exhibited different replication levels over the natural history of chronic HBV infection. Previous studies on the changing patterns of HBsAg, another alternative marker of intrahepatic cccDNA transcription activity, over the natural course of chronic HBV infection, also indicated that HBV exhibited different replication levels through the natural course of chronic infection [26, 29, 41].

Over the natural course of chronic HBV infection, the clinical performance of patients with HBeAg-negative ranges from the “inactive carrier” status to HBeAg-negative hepatitis. Inactive carriers have no or only mild liver tissue damage and with a low incidence of cirrhosis and HCC, but patients with HBeAg-negative hepatitis have sever disease progression and high incidence of cirrhosis [42–44]. Therefore, to distinguish the two stages of HBeAg negative infection is of pivotal significance, because patients with HBeAg negative hepatitis can benefit from therapy. Prior to the commercialization of HBsAg quantitative detection methods, identification of these two stages primarily depends on an HBV DNA cutoff of 2000 IU/ml [3]. However, the significance of the cutoff in clinical practice is controversial [44–46]. For example, in an Italian study, the combination of a single measurement of HBsAg less than 1000 IU/mL and HBV DNA less than 2000 IU/mL developed 94% diagnostic accuracy, 91% sensitivity, 95% specificity, 88% PPV and 97% NPV in identifying “inactive carriers” [47]. However, in a Korean study of 104 patients with genotype C HBe-negative, the combination of a single HBsAg and HBV DNA level developed poor values: 84.6% diagnostic accuracy, 64.5% sensitivity, 93.2% specificity, 80% PPV and 86.1% NPV [48]. These results suggest that it is difficult to accurately predict the disease course with the existing biomarkers. In this study, we found that the serum HBV RNA level was significantly lower in 77 “inactive carriers” than that in the 58 HBeAg-negative hepatitis patients, as follows: median (IQR) 1.70(1.70,2.40) log copies/mL versus 5.24(3.50,5.89), respectively (*P* = 0.000), and, moreover, the serum HBV RNA level provided the best cutoff value to distinguish active patients from patients with HBeAg-negative CHB. Furthermore, the best cutoff value of serum HBV RNA was clearly superior to a single point cutoff of combined HBV DNA (> 3.39 log copies/mL) and HBsAg(> 2.74 log IU/mL) quantification, which allows the identification of active phases of HBeAg-negative CHB patients with a diagnosis accuracy of 73.68%. These results suggest that serum HBV RNA may become a new predictor of HBeAg-negative CHB reactivation, beyond the combination of HBV DNA and HBsAg levels.

The limitation of this study is that our study is a cross-sectional study. A longitudinal study tracking the various stages of chronic HBV infection is warranted. However, because of the progression of many stages over the natural course of chronic HBV infection, such as the EPIA and ENH phases, patients in these phases should receive antiviral therapy to prevent disease progression. Therefore, it is very difficult to design a longitudinal follow-up study to assess the changing pattern of serum HBV RNA levels during the natural course of chronic HBV infection. Thus, our results may provide a basis for understanding the value of serum HBV RNA levels in predicting the natural history of chronic HBV infection.

## Conclusions

In conclusion, as a surrogate marker of intrahepatic cccDNA transcriptional activity, serum HBV RNA varies over the natural course of chronic HBV infection. Serum HBV RNA levels can predict the natural history for chronic HBV-infected individuals, and serum HBV RNA shows superiority in differentiating the ‘HBeAg-negative CHB’ in treatment-naïve HBeAg-negative chronic HBV-infected individuals.

## Abbreviations

ALT: Alanineamino transferase
AST: Aspartate amino transferase
AUROC: Area under the ROC curves
cccDNA: Covalently closed circular DNA
CH: Chronic hepatitis
CHB: Chronic hepatitis B
ENH: HBeAg-negative chronic hepatitis B phase
EPIA: HBeAg-positive immune-active phase
HBeAg: Hepatitis B e antigen
HBsAg: HBV surface antigen
HBV: Hepatitis B virus
ICH: Inactive chronic hepatitis B phase
IT: Immune tolerant
LC: Liver cirrhosis
LOD: The limit of detection
NAs: Nucleosides analogues
NPV: Negative predictive value
pgRNA: pre-genome RNA
PPV: Positive predictive value
qHBsAg: quantitative HBsAg
ROC: Receiver operating characteristic
SAT: Simultaneous amplification testing
