# Correction to: Serum HBV RNA quantification: useful for monitoring natural history of chronic hepatitis B infection

**DOI:** 10.1186/s12876-019-0991-3

**Published:** 2019-05-10

**Authors:** Yayun Liu, Meng Jiang, Jianya Xue, Hongli Yan, Xuesong Liang

**Affiliations:** 1 Department of Infectious Diseases, Changhai Hospital, Second Military Medical University, 168 Changhai Road, Shanghai, 200433 China; 2 Department of Reproductive Medicine Center, Changhai Hospital, Second Military Medical University, 168 Changhai Road, Shanghai, 200433 China

**Correction to: BMC Gastroenterol**

**https://doi.org/10.1186/s12876-019-0966-4**

Following publication of the original article [1], the author reported an error in the “Abstract”, under the “Methods” section. The sentence “inactive chronic hepatitis B(ICH,n = 58) and HBeAg-negative immune reactive hepatitis (ENH,n = 77)” should be change to “inactive chronic hepatitis B(ICH,*n* = 77) and HBeAg-negative immune reactive hepatitis (ENH,*n* = 58).”
